# Infection-Induced Intestinal Dysbiosis Is Mediated by Macrophage Activation and Nitrate Production

**DOI:** 10.1128/mBio.00935-19

**Published:** 2019-05-28

**Authors:** Shuai Wang, Ayah El-Fahmawi, David A. Christian, Qun Fang, Enrico Radaelli, Longfei Chen, Megan C. Sullivan, Ana M. Misic, Jodi A. Ellringer, Xing-Quan Zhu, Sebastian E. Winter, Christopher A. Hunter, Daniel P. Beiting

**Affiliations:** a Department of Pathobiology, School of Veterinary Medicine, University of Pennsylvania, Philadelphia, Pennsylvania, USA; b Department of Microbiology, University of Texas Southwestern Medical Center, Dallas, Texas, USA; c State Key Laboratory of Veterinary Etiological Biology, Key Laboratory of Veterinary Parasitology of Gansu Province, Lanzhou Veterinary Research Institute, Chinese Academy of Agricultural Sciences, Lanzhou, Gansu, China; UC Irvine; University of New Mexico; NIAID, NIH

**Keywords:** gut microbiota, microbiome, *Toxoplasma*, dysbiosis, nitric oxide

## Abstract

Oral infection of C57BL/6J mice with Toxoplasma gondii results in a marked bacterial dysbiosis and the development of severe pathology in the distal small intestine that is dependent on CD4^+^ T cells and interferon gamma (IFN-γ). This dysbiosis and bacterial translocation contribute to the development of ileal pathology, but the factors that support the bloom of bacterial pathobionts are unclear. The use of microbial community profiling and shotgun metagenomics revealed that *Toxoplasma* infection induces a dysbiosis dominated by Enterobacteriaceae and an increased potential for nitrate respiration. *In vivo* experiments using bacterial metabolic mutants revealed that during this infection, host-derived nitrate supports the expansion of Enterobacteriaceae in the ileum via nitrate respiration. Additional experiments with infected mice indicate that the IFN-γ/STAT1/iNOS axis, while essential for parasite control, also supplies a pool of nitrate that serves as a source for anaerobic respiration and supports overgrowth of Enterobacteriaceae. Together, these data reveal a trade-off in intestinal immunity after oral infection of C57BL/6J mice with T. gondii, in which inducible nitric oxide synthase (iNOS) is required for parasite control, while this host enzyme is responsible for specific modification of the composition of the microbiome that contributes to pathology.

## Abstract (precis)

Toxoplasma gondii is a protozoan parasite and a leading cause of foodborne illness. Infection is initiated when the parasite invades the intestinal epithelium, and in many host species, this leads to intense inflammation and a dramatic disruption of the normal microbial ecosystem that resides in the healthy gut (the so-called microbiome). One characteristic change in the microbiome during infection with *Toxoplasma*—as well as numerous other pathogens—is the overgrowth of Escherichia coli or similar bacteria and a breakdown of commensal containment leading to seeding of peripheral organs with gut bacteria and subsequent sepsis. Our findings provide one clear explanation for how this process is regulated, thereby improving our understanding of the relationship between parasite infection, inflammation, and disease. Furthermore, our results could serve as the basis for the development of novel therapeutics to reduce the potential for harmful bacteria to bloom in the gut during infection.

## INTRODUCTION

Toxoplasma gondii is a protozoan parasite that is globally distributed and is a leading cause of foodborne illness (1). Infection is initiated after ingestion of contaminated food or water and results in rapid parasite invasion of the small intestine epithelium and subsequent dissemination throughout the host (2–4). *Toxoplasma* infection has been associated with the development of small intestinal pathology in many different animal species, including humans (5, 6). In certain strains of mice, such as C57BL/6, *Toxoplasma* infection leads to an acute, CD4^+^ T cell-dependent ileitis characterized by an influx of neutrophils and monocytes (7–10), along with increased levels of interferon gamma (IFN-γ) (11, 12), tumor necrosis factor alpha (TNF-α) (11), interleukin 18 (IL-18) (13), IL-22 (14), IL-23 (14), and nitric oxide (NO) (11). These changes in the mucosal environment coincide with extensive disruption of intestinal architecture and physiology (7, 9, 10, 15), which mimics some facets of human inflammatory bowel disease (IBD) (16, 17).

Many enteric parasites, including *Trichuris* (18), *Heligmosomoides* (19), *Giardia* (20), *Blastocystis* (21), *Cryptosporidium* (22), and *Entamoeba* (23), induce marked changes in the structure of the gut microbial community, and there is good evidence that such alterations can contribute to the pathogenesis of these varied infections (24). For example, infection with T. gondii is accompanied by reduced bacterial diversity, a marked expansion of facultative anaerobes such as members of the family Enterobacteriaceae, loss of barrier integrity in the gut, and bacterial translocation that contributes to immune-mediated pathology (9, 16, 17, 24–26). One possible explanation for this dysbiosis and breakdown of commensal containment is that the intestinal barrier is directly compromised during infection, either as a consequence of lytic parasite replication or from toxic by-products of the host immune response, such as reactive oxygen or nitrogen intermediates. Consistent with this notion, recent studies have shown that the production of IFN-γ during infection promotes the loss of Paneth cells which results in reduced levels of antimicrobial peptides, and it has been proposed that this contributes to the dysbiosis (12, 27). These findings, however, do not explain the robust and specific shift in the microbiome during *Toxoplasma* infection to favor Enterobacteriaceae. Moreover, despite the global prevalence of T. gondii and related protozoan parasites, there is a limited understanding of how the T helper type 1 (Th1) immune response to these pathogens shapes microbial community structure in the gut. Several studies have used this model to show that macrophages/monocytes contribute to tissue damage in the gut (8, 15), and interventions that target macrophage activation or recruitment reduce bacterial translocation and restore barrier function (26), yet the cellular mechanisms that mediate these effects remain unknown.

For more than 30 years, it has been appreciated that the ability of IFN-γ to promote macrophage production of NO is an important microbicidal mechanism required for resistance to T. gondii (3, 28–30). Interestingly, chemical inhibition of inducible nitric oxide synthase (iNOS) or genetic deficiency in *Nos2* ameliorates the ileal pathology associated with T. gondii, and it was proposed that NO has direct toxic effects on host cells in the gut that leads to necrosis (11, 28). More recent work using an IL-10-deficient model of spontaneous colitis, or dextran sulfate sodium (DSS) to induce colitis, revealed that host-derived nitrate can directly support a bloom of Enterobacteriaceae bacteria in the colon (31). That report highlighted that local inflammation in the large intestine leads to increased levels of respiratory electron acceptors such as S oxides and N oxides, which serve as a substrate for the growth of facultative anaerobic bacteria (32). The studies presented here investigate the relationship between *Toxoplasma* infection, intestinal inflammation, and bacterial dysbiosis. Using a combination of microbiome profiling and experimentally introduced Escherichia coli strains, we show that host-derived nitrate shapes *Toxoplasma*-induced dysbiosis in the small intestines of C57BL/6J mice. Furthermore, mice deficient in IFN-γ signaling in the monocyte/macrophage population (LysM-Cre STAT1 floxed) produced less nitrate and had a higher parasite burden but displayed a marked reduction in E. coli expansion. Together, these data indicate that the robust Th1 inflammatory response required for local control of T. gondii in C57BL/6J mice also promotes the overgrowth of facultative anaerobes in the ileum, which is known to contribute to bystander inflammation.

## RESULTS

### T. gondii-induced dysbiosis is CD4^+^ T cell dependent and is associated with increased nitrate levels in the ileum.

**FIG 1:**
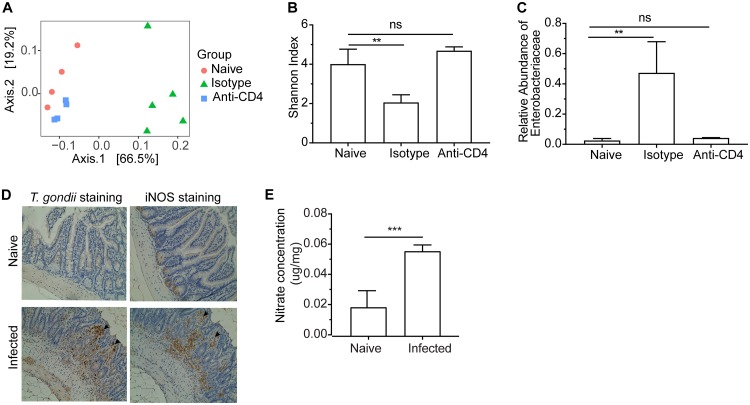
*Toxoplasma-*induced dysbiosis in C57BL/6J mice is dependent on CD4^+^ T cells and is associated with increased nitrate levels in the ileum. (A) Principal coordinates analysis (PCoA) of 16S *rRNA* gene sequencing using weighted UniFrac distance grouped by naive, IgG isotype control and anti-CD4^+^ T cell depletion. (B) Microbiome diversity indicated by Shannon index for different groups. (C) Relative abundance of Enterobacteriaceae bacteria. (D) Representative parasite stained and iNOS stained sections of distal ileum tissues from naive mice and T. gondii-infected mice (day 7) (20× objective). Arrows indicate positive staining of parasites or iNOS. (E) Nitrate concentrations in mucous layers collected from ilea in naive and T. gondii-infected mice (day 7). Results are representative of two independent experiments involving at least four mice per group. Values are means plus standard deviations (SD) of the means (error bars). Statistical significance is indicated as follows: ns, not significant; **, *P* < 0.01; ***, *P* < 0.001.

During T. gondii infection, the accompanying dysbiosis and bacterial translocation contributes to the development of lethal ileitis mediated by CD4^+^ T cells, but whether CD4^+^ T cells directly impact dysbiosis is unclear. Therefore, to investigate the relationship between parasite infection, immune status, and dysbiosis, mice were depleted of CD4^+^ T cells prior to infection, and 16S rRNA maker gene profiling of ileal contents was used to monitor bacterial community structure at the site of parasite infection and pathology. As anticipated (11, 12, 25), oral infection of C57BL/6J mice (note that for clarity, “mice” hereafter refers to C57BL/6J mice unless clearly described as “BALB/cJ mice”) with 50 T. gondii cysts resulted in severe pathology in the ileum by 7 days postinfection (dpi), marked by epithelial tissue necrosis (Fig. S1), a dramatic shift in the ileal microbial community away from naive mice (Fig. 1A), reduced community diversity (Fig. 1B), and increased relative abundance of Enterobacteriaceae (Fig. 1C). At 7 day postinfection, differential abundance analysis comparing naïve mice to infected mice showed a 45-fold increase in *Proteobacteria* (FDR < 0.01) and a 4.8-fold decrease in *Fusobacteria* (FDR < 0.01) (Table S1). The use of a monoclonal antibody to deplete CD4^+^ T cells ameliorated pathology (Fig. S1) and resulted in a microbial community that was nearly indistinguishable from that of naive animals treated with isotype control antibody in terms of community structure (Fig. 1A, squares), diversity (Fig. 1B), and levels of Enterobacteriaceae (Fig. 1C). We further show that this CD4-mediated intestinal dysbiosis did not occur in BALB/cJ mice. After oral infection with 50 T. gondii cysts, no significant changes of gut microbiota structure were observed in BALB/cJ mice (Fig. S2).

10.1128/mBio.00935-19.1 FIG S1 Representative H&E-stained sections of distal ilea of C57BL/6J mice infected with T. gondii and treated with isotype IgG or anti-CD4 (20× objective). Download FIG S1, TIF file, 2.5 MB. Copyright © 2019 Wang et al. 2019 Wang et al. This content is distributed under the terms of the Creative Commons Attribution 4.0 International license.

10.1128/mBio.00935-19.2 FIG S2 Comparison of microbiota structure changes after T. gondii infection between C57BL/6J and BALB/cJ mice. (A) Weight changes after T. gondii infection were recorded on days 0, 2, 4, and 8 postinfection. (B and C) Shannon index of microbiome in C57BL/6J mice (B) and in BALB/cJ mice (C) were calculated based on 16S rRNA profiling data. (D and E) UniFrac (weighted) distance of microbiome between T. gondii-infected mice and naive (day 0) mice were evaluated for C57BL/6J mice (D) and for BALB/cJ mice (E). (F) The competitive index between the E. coli WT strain and the Δ*moaA* mutant strain was determined at day 7 postinfection. Data shown are represented as mean ± SD. ns, not significant; *, *P* < 0.05; ***, *P* < 0.001. Download FIG S2, TIF file, 2.3 MB. Copyright © 2019 Wang et al. 2019 Wang et al. This content is distributed under the terms of the Creative Commons Attribution 4.0 International license.

10.1128/mBio.00935-19.6 TABLE S1 Phyla with differential abundances in naive mice versus T. gondii-infected mice (day 7) (FDR <0.05). Download Table S1, PDF file, 0.01 MB. Copyright © 2019 Wang et al. 2019 Wang et al. This content is distributed under the terms of the Creative Commons Attribution 4.0 International license.

While the ability of CD4^+^ T cell-derived IFN-γ to stimulate macrophage production of iNOS is required for the control of T. gondii (33, 34), previous studies have proposed that iNOS also participates in ileitis (2, 11, 35). In the present study, immunohistochemical staining showed low levels of iNOS in the ilea of uninfected mice, but a marked increase was evident at 7 dpi within the lamina propria at the apical border of the enterocytes and within the core region of the villi of infected C57BL/6J mice (Fig. 1D), often colocalized with sites of parasite replication (Fig. 1D and Fig. S3). Nitrate (NO_3_^−^) is a by-product of iNOS activity and can be generated from the reaction of nitric oxide radicals (NO^·^) with superoxide radicals (O_2_^·–^) (36). Consistent with the levels of iNOS immunoreactivity, the concentration of nitrate in the mucous layer of the ileum was low in naive mice and significantly elevated in infected mice (Fig. 1E). Depletion of CD4^+^ T cells resulted in a marked decrease in the levels of iNOS detected by immunohistochemistry in the infected intestine (Fig. S3).

10.1128/mBio.00935-19.3 FIG S3 Representative parasite stained and iNOS stained sections of distal ileum tissues from the C57BL/6J mice infected with T. gondii (day 7) and treated with isotype IgG or anti-CD4 (20× objective). Arrows indicate positive staining of parasites or iNOS. Download FIG S3, TIF file, 2.9 MB. Copyright © 2019 Wang et al. 2019 Wang et al. This content is distributed under the terms of the Creative Commons Attribution 4.0 International license.

### Nitrate supports overgrowth of E. coli via anaerobic respiration during T. gondii infection.

**FIG 2:**
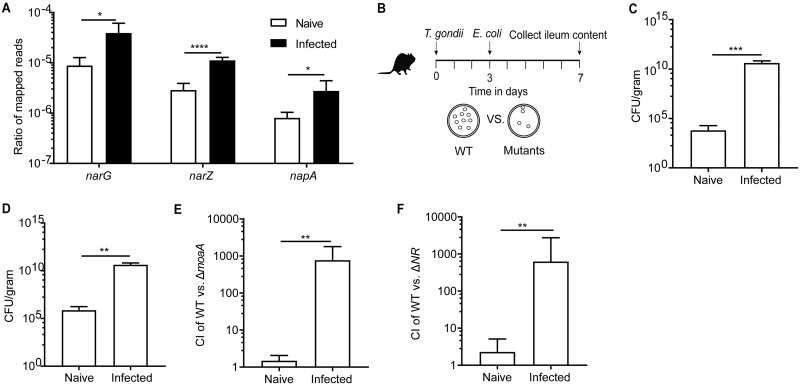
E. coli bloom during *Toxoplasma* infection in C57BL/6J mice is driven in part by nitrate respiration. (A) The ratios of shotgun genomic reads mapped to the reference sequences of the three nitrate reductase-encoding genes (*narG*, *narZ*, and *napA*) in the genome of E. coli. (B) Schematic showing experimental design for E. coli competition experiments. (C) Colony-forming unit (CFU) recovered from the ileum content (day 7) of naive mice and T. gondii-infected mice with E. coli Nissle given on day 3 postinfection. (D) CFU recovered from the ileum content (day 7) of naive mice and T. gondii-infected mice with E. coli Nissle precolonized before T. gondii infection (day −3). (E) Competitive index (CI) between the WT E. coli strain (kanamycin resistant) and the E. coli Δ*moaA* mutant strain (carbenicillin resistant) at day 7 in naive or T. gondii-infected mice that were cocolonized with the two strains on day 3. (F) CI between the WT E. coli strain and the E. coli ΔNR mutant strain at day 7 in naive mice or T. gondii-infected mice that were cocolonized with the two strains at day 3. Results shown are representative of two or more independent experiments involving at least four mice per group. Values are means plus SD. Statistical significance: ns, not significant; *, *P* < 0.05; **, *P* < 0.01; ***, *P* < 0.001; ****, *P* < 0.0001.

Our observation and previous reports (12, 25, 26) that *Toxoplasma* infection is accompanied by an expansion of bacteria that belong to the phylum *Proteobacteria*, together with elevated production of iNOS and nitrate in the intestines of infected mice (Fig. 1D and E), led us to investigate whether nitrate was utilized by *Proteobacteria* as a substrate for anaerobic respiration during T. gondii infection. As a representative member of the family Enterobacteriaceae (within the phylum *Proteobacteria*), Escherichia coli possesses three nitrate reductases encoded by *narGHJI* (*narG*), *narZYWV* (*narZ*), and *napFDAGHBC* (*napA*), which participate in anaerobic respiration through the reduction of nitrate to nitrite (37). To test whether these bacterial genes were enriched after T. gondii infection in C57BL/6J mice, shotgun metagenomics was used to compare global changes in microbial gene content in the ileum. Analysis of the metagenomic sequences from each sample, mapped against E. coli nitrate reductase genes, revealed that genes encoding putative nitrate reductases were significantly enriched in infected mice compared to naive animals (Fig. 2A). These results indicate an increased potential for nitrate utilization during intestinal *Toxoplasma* infection in C57BL/6J mice that correlates with an expansion of Enterobacteriaceae.

To evaluate the importance of nitrate respiration *in vivo* during infection, wild-type (WT) or mutant E. coli Nissle 1917 strains (EcN) were transferred to mice, and bacterial growth was assessed by plating ileal contents under drug selection (Fig. 2B). EcN is widely used for *in vivo* assays to dissect E. coli metabolism during inflammation and is a food-safe probiotic (38). When WT EcN were administered to naive C57BL/6J mice, minimal growth was observed over a 4-day period, with approximately 10^5^ CFU/gram present in ileal contents (Fig. 2C). However, when administered to mice 3 days after *Toxoplasma* infection, WT EcN expanded to greater than 10^10^ CFU/gram in the ileum by 7 dpi (Fig. 2C). A similar trend was observed when mice were precolonized with EcN 3 days prior to parasite infection (Fig. 2D). A common feature shared by the three E. coli nitrate reductases is the incorporation of an essential molybdenum cofactor into the active site. Therefore, to determine whether this pathway was important for the ability of EcN to colonize infected mice, a mutant deficient for the biosynthesis of the molybdenum cofactor (EcN Δ*moaA*) was used in a direct *in vivo* competition experiment with WT EcN (31). In naive mice inoculated with a 1:1 mixture of WT EcN and its isogenic *moaA* mutant, an approximate 1:1 ratio of each strain was recovered from the ileum (Fig. 2E), indicating that in uninfected mice, loss of *moaA* does not alter bacterial fitness. In contrast, at 7 dpi, the WT EcN was recovered from the ileal contents of infected mice at numbers more than 100-fold higher than the *moaA* mutant (Fig. 2E). Interestingly, the competitive experiment in ileitis-resistant BALB/cJ mice phenocopied the results in naive C57BL/6J mice (Fig. S2F). Taken together, these data show that molybdenum cofactor-dependent processes are not required for establishment in the ileum during homeostasis but are critical for expansion during *Toxoplasma*-induced inflammation in C57BL/6J mice.

E. coli produces three nitrate reductases, three TMAO reductases, two DMSO reductases, and three formate dehydrogenases—all of which are molybdoenzymes and require molybdenum cofactor for their function (39). Given the potent induction of iNOS and nitrate during infection (Fig. 1), we hypothesized that nitrate respiration would be the primary molybdenum cofactor-dependent pathway required for E. coli expansion in the T. gondii-infected intestines of C57BL/6J mice. To test this hypothesis, infected mice were administered a 1:1 mixture of a WT EcN or EcN strain engineered to be deficient in all three nitrate reductases (Δ*narG* Δ*narZ* Δ*napA*; ΔNR mutant). In naive mice, low but comparable levels of both strains were recovered (Fig. 2F). In contrast, in infected mice, the ΔNR mutant showed a marked defect in expansion compared to WT EcN (Fig. 2F), which phenocopied results with the Δ*moaA* mutant (Fig. 2E). Similar results were obtained when the ΔNR mutant and WT EcN were used to colonize separate groups of mice (fold change of WT/ΔNR = 77.13; *P* = 0.0016), indicating that the growth defect of the mutant observed in the direct competition experiments is not a consequence of interactions between strains. These data establish that during *Toxoplasma* infection, bacterial nitrate reductase activity is required to support E. coli expansion in the small intestine.

### Chemical inhibition of host iNOS reduces growth benefit from nitrate respiration.

**FIG 3:**
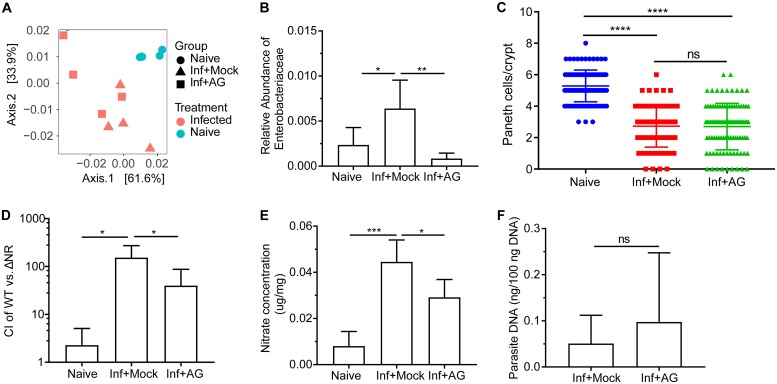
Inhibition of iNOS abrogates the growth advantage from nitrate respiration after *Toxoplasma* infection in C57BL/6J mice. (A) PCoA of 16S rRNA gene sequencing results of ileum content using weighted UniFrac distance grouped by mice and treatment. The groups were naive mice (Naive), T. gondii-infected mice treated with PBS (Inf+Mock), and T. gondii*-*infected mice treated with aminoguanidine (AG) (from day 0 to day 7) (Inf+AG). (B) Relative abundance of Enterobacteriaceae bacteria in the three groups. (C) Quantification of Paneth cells per crypt in naive mice and T. gondii-infected mice mock treated or treated with AG. (D) The CI between the E. coli WT strain and the ΔNR mutant strain was determined at day 7. (E) Nitrate concentrations in the ileal mucous layers. Mice were mock treated or treated with AG at start (day 3) of competition experiments. (F) Parasite burden was quantified from distal ileum. Results are representative of two independent experiments (D to F) involving at least four mice per group. Data shown are represented as means plus SD. Statistical significance: ns, not significant; *, *P* < 0.05; **, *P* < 0.01; ***, *P* < 0.001; ****, *P* < 0.0001.

There are multiple potential cellular sources of nitrate produced by distinct NOS enzymes. To determine whether iNOS was required for the growth advantage of E. coli in the ileum, C57BL/6J mice infected with T. gondii were treated with a selective iNOS inhibitor, aminoguanidine hydrochloride (AG). In mice treated with AG, infection with *Toxoplasma* still resulted in marked dysbiosis (Fig. 3A), but expansion of Enterobacteriaceae was blunted (Fig. 3B). Previous reports suggest a role for Paneth cells in *Toxoplasma*-induced dysbiosis (12, 27). Therefore, one possible explanation for the effect of AG on the relative abundance of Enterobacteriaceae is that reduced iNOS levels prevented Paneth cell loss during infection. To address this possibility, Paneth cells were counted in naive and infected mice that had been treated with AG or not treated with AG. Consistent with prior studies, infection was associated with a significant reduction in Paneth cell number, but AG treatment did not impact this phenotype (Fig. 3C). Moreover, when infected mice were inoculated with a mixture of the WT EcN and the ΔNR mutant at 3 dpi, AG treatment reduced the competitive advantage of WT EcN over the triple mutant (Fig. 3D). This short-term treatment (days 3 to 7) with AG, however, did significantly reduce levels of nitrate in the ileal mucous layer but did not significantly alter parasite burden (Fig. 3E and F, respectively). Collectively, these data highlight that induction of iNOS in the ileum during infection contributes to overgrowth of Enterobacteriaceae that are dependent on bacterial nitrate respiration.

### Macrophages contribute to the dysbiosis via STAT1-mediated iNOS production.

**FIG 4:**
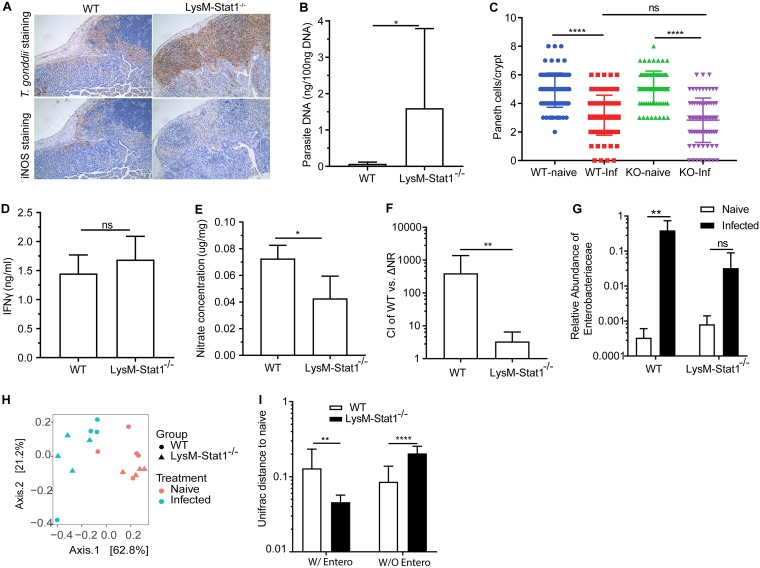
Nitrate-dependent expansion of E. coli during infection is reduced in LysM-Stat1^−/−^ mice. (A) Representative sections stained for parasite or iNOS in ilea of WT mice and LysM-Stat1^−/−^ mice 7 days postinfection (20×objective). Peyer’s patches and adjacent villi are shown. (B) Parasite burden. (C) Quantification of Paneth cells per crypt. (D and E) IFN-γ levels of ileum tissues (D) and nitrate concentrations in muocus layer (E) were detected. (F) Competition index (CI) between the E. coli WT strain and the ΔNR triple mutant revealed in competition experiments in WT mice and LysM-Stat1^−/−^ mice. (G) Relative abundance of Enterobacteriaceae bacteria in these mice. (H) PCoA of 16S rRNA gene sequencing results of ileum content using weighted UniFrac distance grouped by WT mice (naive and infected) and LysM-Stat1^−/−^ mice (naive and infected). (I) Weighted UniFrac distance of ileum microbiomes in T. gondii-infected WT and LysM-Stat1^−/−^ mice to the ileum microbiomes in corresponding naive mice. The distances were calculated based on microbiota compositions with Enterobacteriaceae bacteria (W/Entero) or without Enterobacteriaceae bacteria (W/O Entero). Results are representative of two or more independent experiments (B to F) involving at least four mice per group. Data shown are represented as means plus SD. Statistical significance: ns, not significant; *, *P* < 0.05; **, *P* < 0.01; ****, *P* < 0.0001.

Given that IFN-γ-mediated activation of STAT1 promotes macrophage expression of iNOS during T. gondii infection, we hypothesized that these cells would be pivotal to the modification of microbiota composition during inflammation. To test this hypothesis, mice expressing Cre recombinase driven by the macrophage/granulocyte-specific M lysozyme gene (LysM-Cre) were crossed with mice that contain floxed alleles of STAT1 (LysM-Stat1^−/−^). In accordance with previous studies that suggest the critical roles of STAT1 in the production of iNOS (40, 41), macrophages from these mice are unable to respond to IFN-γ via STAT1 and show a reduced capacity to produce iNOS (Fig. S4 and Text S1). After oral infection, LysM-Stat1^−/−^ mice had a profound increase in parasite burden associated with severe necrosis of the small intestinal epithelium that extended into the jejunum and duodenum, and extensive cellular infiltration evident in the lamina propria (Fig. 4A and B). No significant differences in Paneth cell loss per crypt were observed between the infected WT and LysM-Stat1^−/−^ mice (Fig. 4C), but increased disruption of crypt structure was observed in the LysM-Stat1^−/−^ mice, presumably due to increased parasite replication. Importantly, this increased susceptibility did not appear to be a secondary consequence of reduced NK and T cell responses, as the IFN-γ levels were not significantly changed in the LysM-Stat1^−/−^ mice (Fig. 4D). Rather, in the LysM-Stat1^−/−^ mice, the infection-induced iNOS in the lamina propria and the levels of nitrate in the mucous layer were significantly reduced (Fig. 4A and E). Furthermore, when the ability of WT EcN and ΔNR mutants to colonize WT and LysM-Stat1^−/−^ mice was evaluated, a marked reduction in the competition index was observed (Fig. 4F). Thus, not only is STAT1 signaling in macrophages required for parasite control, but it is also necessary for this infection-driven dysbiosis.

10.1128/mBio.00935-19.4 FIG S4 The levels of expression of iNOS in macrophages of LysM-Stat1^−/−^ mice were reduced. Flow cytometry was used to analyze the expressions of iNOS, MHCII, and CD11b in large peritoneal macrophages (LPMs). Data shown are represented as means ± SD. ns, not significant; *, *P* < 0.05; **, *P* < 0.01; ***, *P* < 0.001. Download FIG S4, TIF file, 2.1 MB. Copyright © 2019 Wang et al. 2019 Wang et al. This content is distributed under the terms of the Creative Commons Attribution 4.0 International license.

10.1128/mBio.00935-19.7 TEXT S1 Detection of iNOS expression in macrophages of LysM-Stat1^−/−^ mice. Download Text S1, PDF file, 0.1 MB. Copyright © 2019 Wang et al. 2019 Wang et al. This content is distributed under the terms of the Creative Commons Attribution 4.0 International license.

Analysis of microbiome composition in the ileum using 16S rRNA gene sequencing revealed that, as expected, infection of WT mice resulted in a shift in community composition which was characterized by a dramatic increase in the relative abundance of Enterobacteriaceae (*P* < 0.05). In contrast, LysM-Stat1^−/−^ mice exhibited a modest shift in Enterobacteriaceae that was approximately 12-fold lower than WT mice (Fig. 4G), whereas naive WT and LysM-Stat1^−/−^ mice had similar baseline levels of Enterobacteriaceae (*P* = 0.097) (Fig. S5). Interestingly, loss of STAT1 in macrophages failed to completely protect mice from dysbiosis, with an obvious shift in microbiome composition still evident after infection (Fig. 4H), albeit reduced in comparison to infected WT mice. When Enterobacteriaceae were excluded from this analysis, infected LysM-Stat1^−/−^ mice showed a greater distance from naive mice (Fig. 4I). These data show that the loss of STAT1 signaling in macrophages does not prevent dysbiosis but rather specifically impairs the extent to which Enterobacteriaceae contribute to this dysbiosis and that during infection, IFN-γ signaling and nitric oxide production in macrophages are key events that lead to a specific shift in microbiome composition.

10.1128/mBio.00935-19.5 FIG S5 Relative abundances of Enterobacteriaceae in naive WT and LysM-Stat1^−/−^ mice. Data shown are represented as means ± SD. Download FIG S5, TIF file, 0.3 MB. Copyright © 2019 Wang et al. 2019 Wang et al. This content is distributed under the terms of the Creative Commons Attribution 4.0 International license.

## DISCUSSION

The data presented here highlight that an oral infection with T. gondii in C57BL/6J mice results in a marked increase in the available pool of nitrate in the small intestine, which in turn supports the growth of E. coli—a key event associated with bacterial translocation and development of systemic pathology (12, 25). The extent to which nitrate respiration provides a growth benefit for pathogenic microbes was illustrated by *in vivo* experiments with E. coli Nissle strain metabolic mutants and the ability of the NO synthase inhibitor, aminoguanidine, to block the bloom of Enterobacteriaceae during *Toxoplasma* infection. While these data do not rule out a direct contribution of NO to cell death, they provide an alternative explanation for previous reports that highlighted the contribution of IFN-γ and iNOS to gut pathology during toxoplasmosis (11, 28). Taken together with previous studies showing that nitrate production also supports E. coli growth during chemically induced colitis and in spontaneous colitis that develops in IL-10-deficient mice (31), there appears to be a broad capacity for E. coli to leverage substrates made during inflammatory responses that occur during infectious and noninfectious insults in either the large or small intestine.

Previous studies have established the important contribution of activated macrophages and iNOS to the development of the T. gondii-induced ileitis in C57BL/6J mice (11). The reduced expansion of Enterobacteriaceae observed in LysM-Stat1^−/−^ mice resembles results reported for *Toxoplasma*-infected mice deficient in CCR2 or treated with antibody to TNF-α (26). Given that CCR2 and TNF-α are essential for the recruitment and activation of macrophages to express iNOS, these data support the conclusion that macrophage-derived NO is a critical driver of dysbiosis in this experimental model. Interestingly, in BALB/cJ mice infected with T. gondii, neither intestinal dysbiosis nor a nitrate-dependent growth benefit for E. coli was observed. It remains to be determined what underlies the differential susceptibility of C57BL/6J and BALB/cJ mice to dysbiosis and whether this contributes to the reduced susceptibility of BALB/c mice to other models of intestinal inflammation (42). However, compared to C57BL/6J mice, macrophages from BALB/cJ mice produce less iNOS following stimulation with LPS and IFN-γ (43) or upon infection (44). Together, these data highlight that qualitative differences in the macrophage response can profoundly influence the development of enteric dysbiosis. Nevertheless, IFN-γ-dependent, iNOS-independent mechanisms of resistance mediated by guanylate binding proteins and immunity-related GTPases are critical to restrict T. gondii in human and mice (45, 46). Thus, while the infection of LysM-Stat1^−/−^ mice is associated with reduced iNOS and nitrate production, the uncontrolled parasite replication in the small intestines of these mice also formally establishes the importance of the IFN-γ-dependent, STAT1-mediated antimicrobial effector functions in macrophages for the control of T. gondii *in vivo*. These data are informative in several ways: first, they suggest that direct damage to the gut caused by lytic parasite replication is not sufficient to cause dysbiosis, and second, they highlight that Paneth cell loss, which has been previously considered a trigger for dysbiosis (12, 27) but which was unaffected by the loss of *Stat1* in macrophages, may not be sufficient to allow unchecked expansion of Enterobacteriaceae.

Our study demonstrates that myeloid-derived nitrate is critical for expansion of E. coli during infection, but we have not determined the specific subset(s) of macrophages or monocytes responsible. Other cell types, such as epithelial cells (44, 45) and smooth muscle cells (46), have also been reported to express iNOS during intestinal diseases, and additional studies will be required to determine the relative contributions of these different sources of NO in the gut. Nevertheless, the ability to modulate nitrate levels, either through sustained AG treatment or depletion of STAT1 in myeloid cells and thereby influence microbiome composition during an infection-induced ileitis, highlights potential avenues for precision editing of the enteric microbiome. We observed that a limited duration (4 days) of AG treatment had only a modest effect on the growth of adoptively transferred E. coli Nissle, but this could be a consequence of the concentration of AG in the gut. Importantly, neither sustained AG treatment nor STAT1 depletion in myeloid cells completely prevented the global dysbiosis. One possible explanation for this phenomenon is that the loss of Paneth cells triggers dysbiosis, and by inhibiting E. coli expansion, we allowed other members of the gut microbiome to compete for resources and grow independent of nitrate. In addition, although mutant EcN lacking either *moaA* or nitrate reductases were less efficient at colonization than WT EcN, they still reached levels typically greater than 10^10^ CFU/gram in the ileum, suggesting that other substrates support E. coli expansion during *Toxoplasma* infection. Indeed, studies in other models of gut inflammation have shown that other electron donors and acceptors, such as formate (47) and oxygen (48), can be exploited by commensal bacteria to allow growth (49, 50). Additional metabolic profiling will be required to determine the full range of substrates and immune cell types that shape dysbiosis during parasite-induced inflammation.

Since its original description as a model to study Th1-mediated gut pathology (16, 17), oral infection of C57BL/6J mice with T. gondii has served as a tractable system to dissect the events that lead to CD4^+^ T cell-mediated inflammation in the gut and the impact of the local microbiota on these events. There is increased recognition of direct cross talk between the immune system and the microbiome, through the production of IgA that coats pathobionts in the gut (46) and the generation of microbe-derived metabolites such as secondary bile acids and short-chain fatty acids that have profound effects on T cell populations in the gut (47). The results from this study provide an illustration of how Th1 inflammation can lead to dramatic changes in the availability of nitrate in the mucous layer which support growth of E. coli, and complement other reports that mucus can serve as a substrate that supports the expansion of Akkermansia muciniphila (48). Collectively, these data highlight that rather than considering dysbiosis as a driver of inflammation, it could be used as an indicator of the type of pathological processes present in the gut. Furthermore, these data show that Th1 responses required to produce potent effector molecules such as NO in order to kill protozoan parasites can potentially come at the expense of commensal containment.

While little is known about the interactions between intestinal parasites and commensal bacteria, our observation that Th1-induced nitrate production can promote dysbiosis raises questions about how widely conserved this mechanism is across diverse parasites and whether such transkingdom interactions can impact the outcome of infection. Interestingly, dysbiosis characterized by increased prevalence of Enterobacteriaceae has also been reported during infection with *Giardia* (49) and Entamoeba histolytica (50, 51). Rather than simply inducing inflammation, the E. coli bloom during *Entamoeba* infection produces malate dehydrogenase that protects E. histolytica from oxidative damage by the host (52). Remarkably, physical interaction with E. coli or other *Proteobacteria* was shown to be required for optimal hatching of eggs from the parasitic nematode Trichuris muris (53). Nematodes like T. muris induce potent Th2 responses and are often associated with immune suppression, and there is mounting evidence that this type of immune response influences, and is influenced by, the microbiome. For example, infection with the nematode Trichinella spiralis results in decreased relative abundance of *Turicibacteraceae* and increased *Lactobacillacaeae* in the small intestine (54). Infection with Heligmosomoides polygyrus also results in increased relative abundance of *Lactobacillaceae* (55), and the potent immune suppression exhibited during H. polygyrus infection is mediated by the microbiome (56, 57). In these examples, infection-induced alterations in the bacterial community may contribute to parasite success, and consequently, a better understanding of how microbiota-host interactions influence the outcome of parasite infections remains an important challenge.

## MATERIALS AND METHODS

### Mice.

Female 7-week-old C57BL/6J and BALB/cJ mice were purchased from Jackson Laboratory and maintained for 2 to 3 weeks in the animal facility at the University of Pennsylvania before any experiments. STAT1^flox^ mice were generated as previously described (58). LysM-Cre mice (B6.129P2-*Lyz2tm1(cre)Ifo*/J; MGI: 1934631) were obtained from Jackson Laboratory and were crossed with STAT1^flox^ in-house to generate the STAT1^fl/fl^ × LysM-Cre (LysM-Stat1^−/−^) mice. Age- and sex-matched STAT1^fl/fl^ mouse littermates served as controls for the experiments with LysM-Stat1^−/−^ mice. Genotypes of all the homozygotes were identified by PCR in accordance with Jackson Laboratory’s protocols. All mice were bred and maintained under pathogen-free conditions at an American Association for the Accreditation of Laboratory Animal Care-accredited animal facility at the University of Pennsylvania and housed in accordance with the procedures outlined in the *Guide for the Care and Use of Laboratory Animals* (59).

### Parasite infections.

T. gondii cysts (type II, ME49 strain) used in experiments were obtained from homogenized brains of chronically infected CBA mice that were inoculated 1 to 3 months previously with 20 cysts by intraperitoneal (i.p.) injection. All mice were randomly assigned into groups before any experiments (day 0) and then infected orally with an average of 50 cysts in an inoculum of 0.2 ml phosphate-buffered saline (PBS) (pH 7.4) by gavage. Naive mice within each experiment were gavaged with an equal volume of PBS as vehicle control. At days 7 postinfection, mice were euthanized to collect tissues and bacterial samples. All procedures were performed in accordance with the guidelines of the University of Pennsylvania Institutional Animal Care and Use Committee.

For measuring parasite burden, approximately 1 cm of distal ileum was removed and flushed using PBS. Parasite DNA was quantified by real-time PCR as previously described (60). Briefly, DNA was extracted from the tissues using DNeasy Blood & Tissue kit (Qiagen, USA) and quantified by Qubit 3.0 fluorometer (Thermo Fisher Scientific). Primers (Invitrogen; forward primer, 5′-TCCCCTCTGCTGGCGAAAAGT-3′; reverse primer, 5′-AGCGTTCGTGGTCAACTATCGATTG-3′) for the T. gondii B1 repeat region and standard curves were used to quantify the amount of parasite DNA from 100 ng tissue DNA, using Power SYBR Green PCR master mix (Applied Biosystems) and an Applied Biosystems ViiA 7 real-time PCR system.

### E. coli strains and *in vivo* competition assay.

The generation of EcN mutants that are individually deficient in the molybdenum cofactor-encoding gene (EcN Δ*moaA*; SW1029) or the three nitrate reductase-encoding genes (EcN ΔNR*;* SW930) have been described in detail previously (31). Strains were tagged with the low-copy-number plasmids pWSK29 and pWSK129 to confer resistance to carbenicillin and kanamycin, respectively (61). *In vitro* anaerobic growth assays were performed with each of the indicated E. coli strains as described previously (31) to guarantee the growth benefit from nitrate respiration in the presence of nitrate sources. All strains were routinely grown aerobically in lysogeny broth (LB) or on LB plates (Difco; Lennox) plates at 37°C. At day 3 after T. gondii infection, each mouse was gavaged with 200 μl LB inoculum that contained an equal number (5** × **10^8^ CFU) (1:1) of WT and mutant strains (Δ*moaA* or ΔNR). At day 7, ileum lumen content was harvested, weighed, and plated on LB agar plates containing strains with kanamycin or carbenicillin resistance (working concentration, 100 μg/ml), respectively. The competitive index (CI) was calculated by normalizing the ratio of recovered wild-type bacteria to mutant bacteria (output ratio) to the respective ratio in the inoculum (input ratio). For the precolonization experiments, mice were gavaged with 1** × **10^9^ CFU (200 μl in LB broth) of either WT or mutants 3 days before T. gondii infection. The collected ileum content was weighed and plated on the selective kanamycin and carbenicillin LB agar plates, respectively.

### CD4^+^ T cell depletion.

Mice were injected intraperitoneally with 0.5 mg of anti-CD4 MAb (GK1.5, BioXcell) in 200 μl PBS on the day prior to T. gondii infection. Control mice were injected with 0.5 mg of isotype IgG2b (BioXcell) in the same manner. The efficiency of CD4^+^ T-cell depletion was assessed by flow cytometric analysis at day 7 as previously described (62), and depletion was >95% for CD4^+^ T cells in the spleens that were isolated from the mice.

### Histology and immunohistochemistry.

Distal ileum tissues were cut, immediately fixed in 10% formalin solution (Sigma-Aldrich), embedded in paraffin, and sectioned at 10 μm. The obtained sections were stained with hematoxylin and eosin (H&E) or Alcian blue for histological evaluations. To count Paneth cells, 20 crypts in each section were randomly selected and evaluated only if aligned along the longitudinal axis such that the lumen of the crypt could be seen along its length. Only crypts that have a clear structure were counted. Paneth cells were identified on the basis of their morphology of cytoplasmic eosinophilic granules and a basolateral nucleus at the base of the intestinal crypt. To detect the presence of parasites and iNOS signals, sections were stained by an immunohistochemistry method using rabbit polyclonal anti-ME49 sera or rabbit polyclonal anti-iNOS antibody (catalog no. ab15323; abcam), goat anti-rabbit biotinylated antibody (catalog no. BA-1000; Vector Laboratories), and avidin-biotin complex interaction systems (ABC HRP kit; Vector Laboratories) according to the manufacturer’s instructions. Images were collected on an Olympus BX61 microscope (Olympus, Japan) and analyzed using NISElement (Nikon, Japan). Histological sections were evaluated by a board-certified veterinary pathologist.

### Aminoguanidine treatment.

For *in vivo* competition experiments using EcN strains (Fig. 3D to F), mice were maintained on either filter-sterilized water (mock treatment) or a filter-sterilized solution of aminoguanidine hemisulfate salt (Sigma-Aldrich) (1 mg/ml) in water beginning on the same day as inoculation with EcN (3 days postinfection with T. gondii). In order to avoid any potential direct effect from aminoguanidine on gut microbiota structure, intraperitoneal injection (2 mg/mouse daily, dissolved in PBS) was used for 16S rRNA profiling experiments (Fig. 3A to C) from day 0 to the end of the experiment, and the control group included mice administered PBS (mock).

### 16S rRNA profiling and shotgun metagenomics.

Ileal contents and fecal samples were collected and stored at −80°C. Genomic DNA was purified using the PowerSoil DNA Isolation kit (MO BIO Laboratories, Carlsbad, CA) following the manufacturer’s recommendations. For 16S rRNA gene profiling, a dual-index amplicon sequencing method was employed for PCR amplification of the V4 region of the 16S *rRNA* gene (63), and sequencing was conducted on an Illumina MiSeq instrument (San Diego, CA) to generate 250-bp paired-end reads. Raw reads were filtered to remove sequences with average Phred quality scores of ≤20 using Quantitative Insights into Microbial Ecology (QIIME) (64) with filtering options (-q 20 -p 0.75 -r 3). Homopolymers of >10 bp in length and sequences of <248 bp and >255 bp were removed using mothur (65). Chimeric sequences were identified and removed by usearch61 against the representative 16S sequences of SILVA128 (97_otus_16S.fasta). Quality-controlled sequences were then clustered against the SILVA128 database (SILVA_128_QIIME_release) using the open-reference OTU picking as implemented in QIIME with default parameters. Singletons were removed from the OTU tables. Analysis of OTU tables was carried out using the R statistical environment and the Phyloseq (66) package. The OTU table was rarefied to 4,700 sequences per sample for the CD4 depletion experiment, 4,500 sequences for the AG treatment experiment, 9,000 sequences for experiments of LysM-Stat1^−/−^ mice, and 6,800 sequences for experiments comparing the microbiota structure of C57BL/6J mice and BALB/cJ mice. Alpha diversity was evaluated using Shannon index. Principal coordinates analysis (PCoA) method was based on the weighted UniFrac distance after removal of rare OTUs (>0.05% in least two samples). Deseq2 (67) was used to perform differential analysis at a phylum level (>0.05% in least two samples).

Metagenomic sequencing libraries were prepared using Illumina Nextera XT with 1 ng of DNA. Sizing and quantification of libraries were conducted using a Tapestation 4200 (Agilent) and Qubit 3.0 fluorometer (Thermo Fisher Scientific), respectively. Equimolar amounts of each library were pooled and sequenced on an Illumina NextSeq 500 instrument to produce 150-bp paired-end sequences. Sequencing adapters and low-quality reads were trimmed and filtered by Trimmomatic (v0.36) (leading:3 trailing:3 slidingwindow:4:15 minlen:36). High-quality reads were mapped to the mouse reference genome (GRCm38), using Bowtie2 v2.3.4.1 (--very-sensitive), and aligned reads were removed using SamTools (68). After host contamination filtering, the resulting high-quality reads were mapped to the reference sequences of nitrate reductases of E. coli (GenBank accession no. NC_000913.3).

### Determination of cytokine and nitrate concentrations.

For cytokines, the levels of IFN-γ in ileum tissue were determined as described previously (25). Briefly, ileum samples (∼1 cm^2^) were flushed thoroughly with sterile PBS, cut longitudinally, and cultured in 24-well plates containing 0.5 ml of RPMI 1640 (Invitrogen Life Technologies) with penicillin (100 I.U./ml) and streptomycin (100 μg/ml) (Sigma-Aldrich). After 24 h at 37°C, supernatants were harvested and stored at −80°C. IFN-γ concentrations were determined by ELISA (BD Biosciences). The lumenal content of the ileum was removed, and the ileum mucous layer was collected and weighed. Ileal mucus was extracted with 0.2 ml ultrapure water (Gibco), and larger particles were removed by centrifugation at 20,000 × *g* for 10 min at 4°C. Nitrate concentrations were measured using a modified Griess reagent [400 mg vanadium(III) chloride, 200 mg sulfanilamide, 10 mg *N*-(1-naphthyl) ethylenediamine dihydrochloride in 150 ml of 1 M HCl] (Sigma-Aldrich). As water-soluble proteins would be precipitated when the acid reagent was added into the mucous supernatants, an equal volume of 1 M HCl was added to the samples to remove any potential precipitation prior to starting the Griess reaction. The precipitated particles were removed by centrifugation at 20,000 × *g* for 10 min at 4°C. The supernatant was mixed with an equal volume of the modified Griess reagent for 8 h at room temperature. The absorbance at 540 nm was measured photometrically. Nitrite concentrations were calculated from standard curves. This procedure measures nitrate together with nitrite.

### Statistical analysis.

Statistical analysis was performed using Prism 7 software (GraphPad Software, La Jolla, CA). The statistical significances of competitive indices were determined using Mann-Whitney U test. Other statistical significance was determined by two-tailed unpaired Student’s *t* test for independent samples. *P* values were adjusted by Benjamini-Hochberg (BH) method for multiple hypothesis tests in differential abundance analyses of 16S rRNA profiling data sets. Values of *P* < 0.05 or FDR <0.05 were considered significant.

### Availability of data.

The raw 16S rRNA sequences and sample information are deposited in NCBI Sequence Read Archive (SRA) database, under project accession no. PRJNA522275 and study accession no. SRP185846.
