# Supplementary figures and images for: Infection-Induced Intestinal Dysbiosis Is Mediated by Macrophage Activation and Nitrate Production
Source: mBio. 2019 May 28;10(3):e00935-19. doi: 10.1128/mBio.00935-19 (PMC6538788; doi:10.1128/mBio.00935-19)

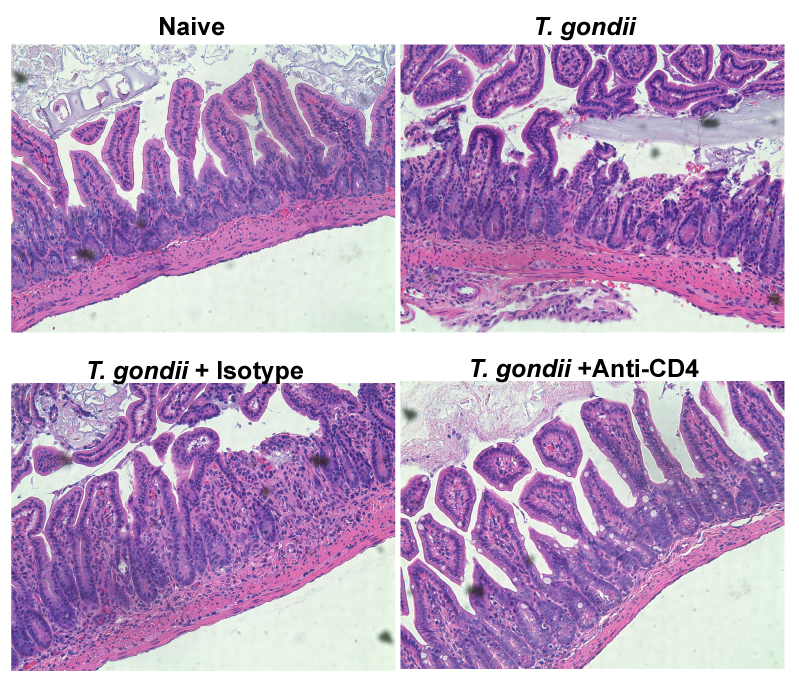

Supplement: FIG S1 [file mBio.00935-19-sf001.tif]

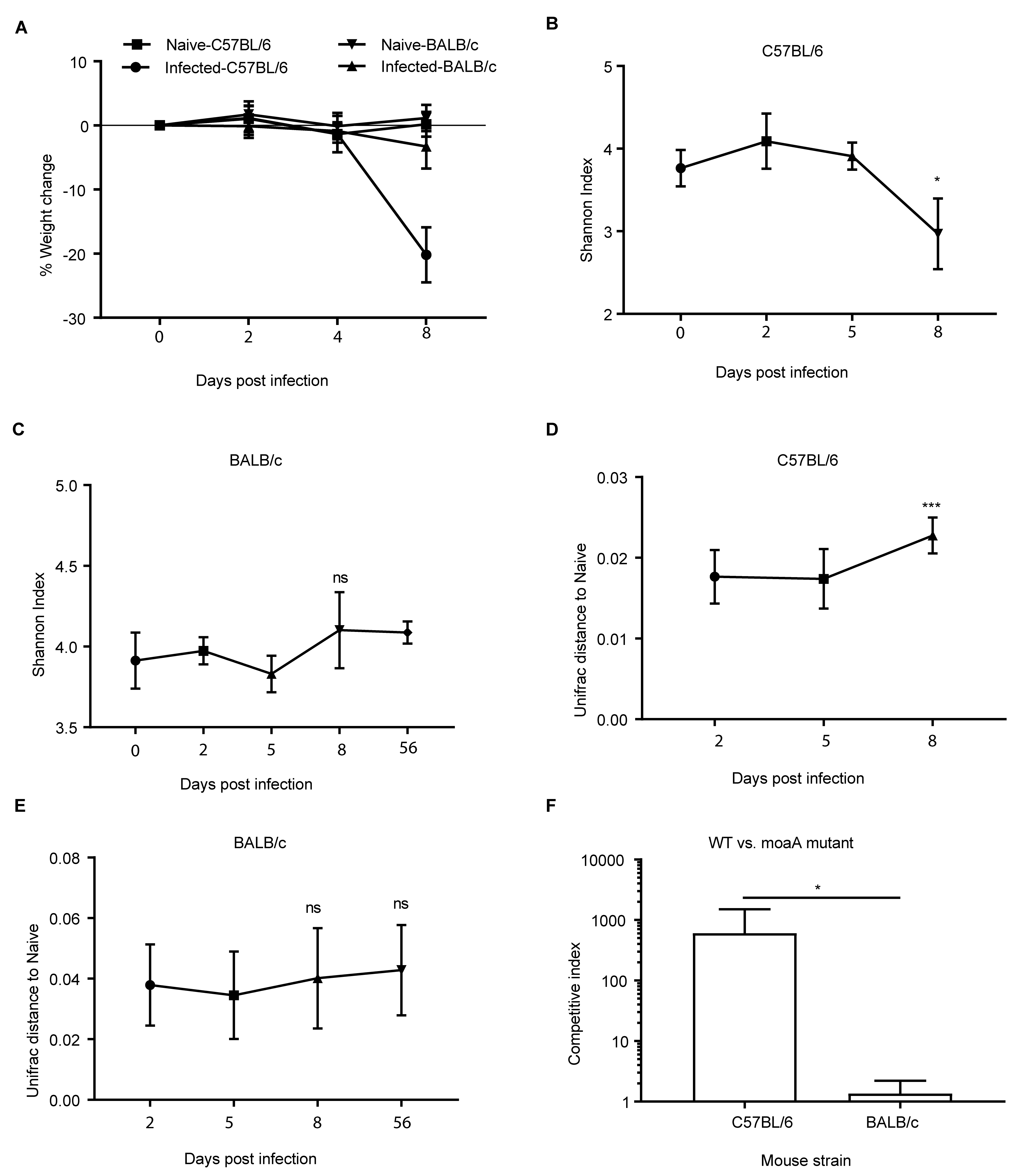

Supplement: FIG S2 [file mBio.00935-19-sf002.tif]

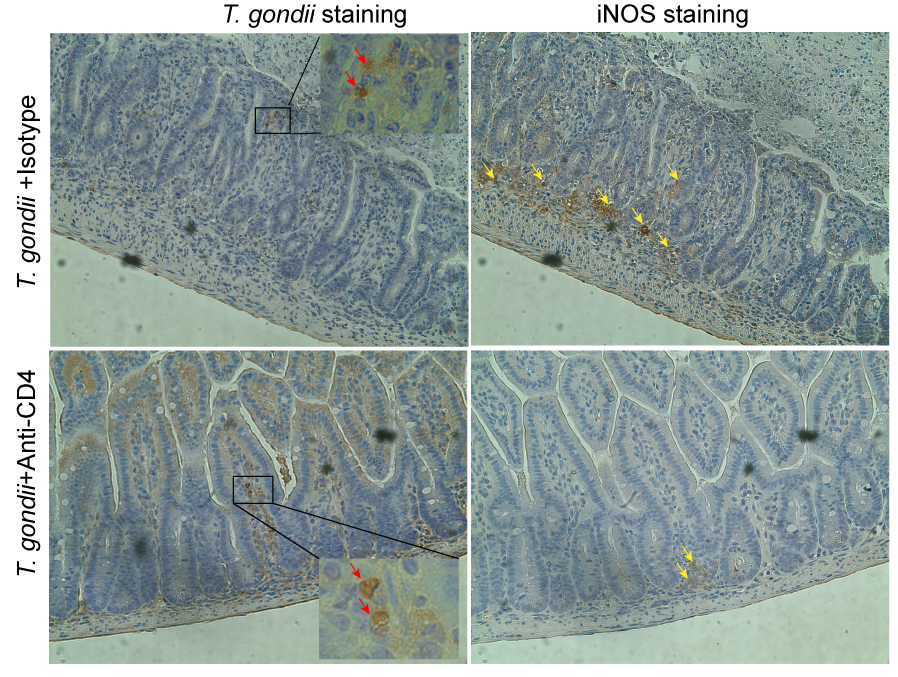

Supplement: FIG S3 [file mBio.00935-19-sf003.tif]

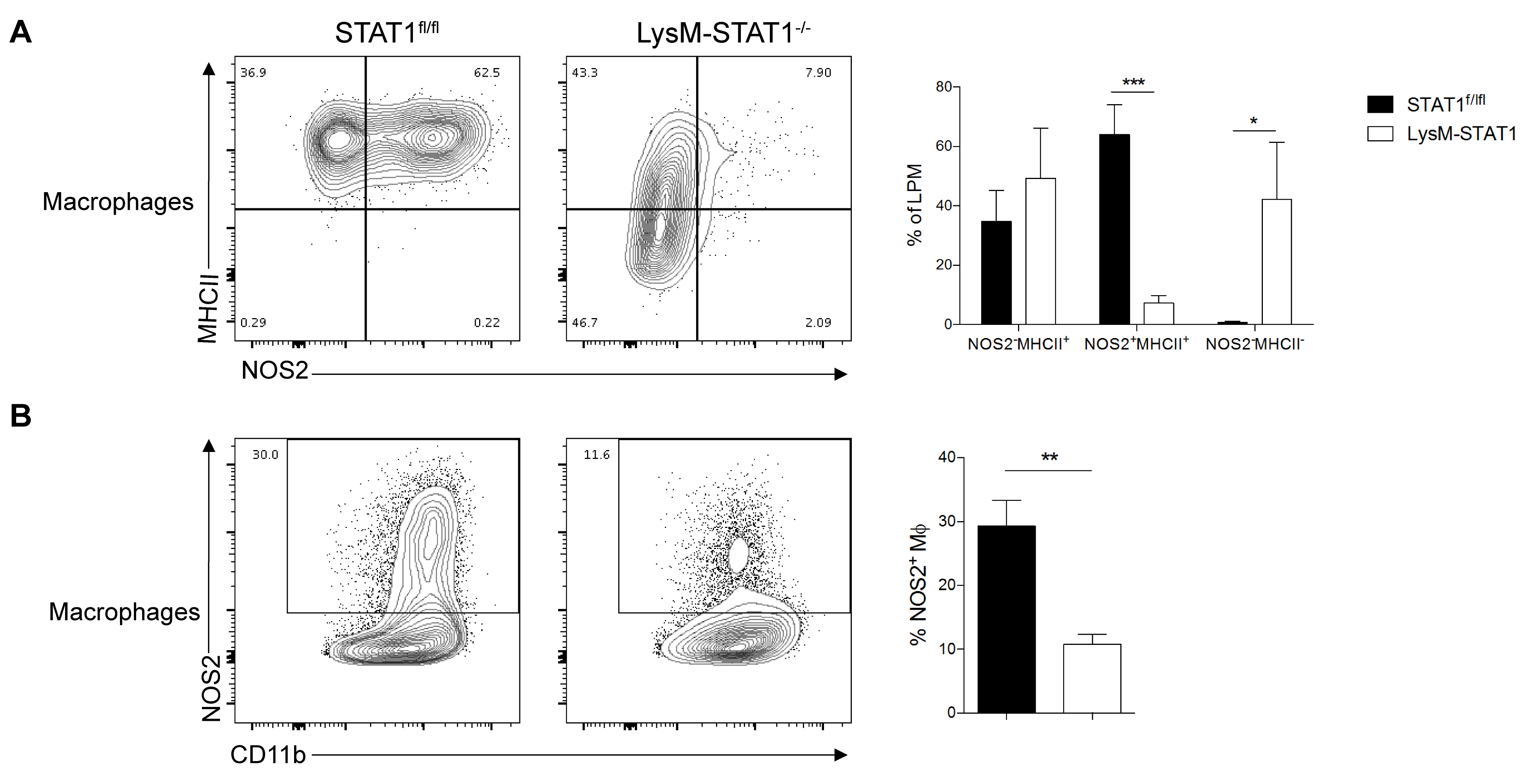

Supplement: FIG S4 [file mBio.00935-19-sf004.tif]

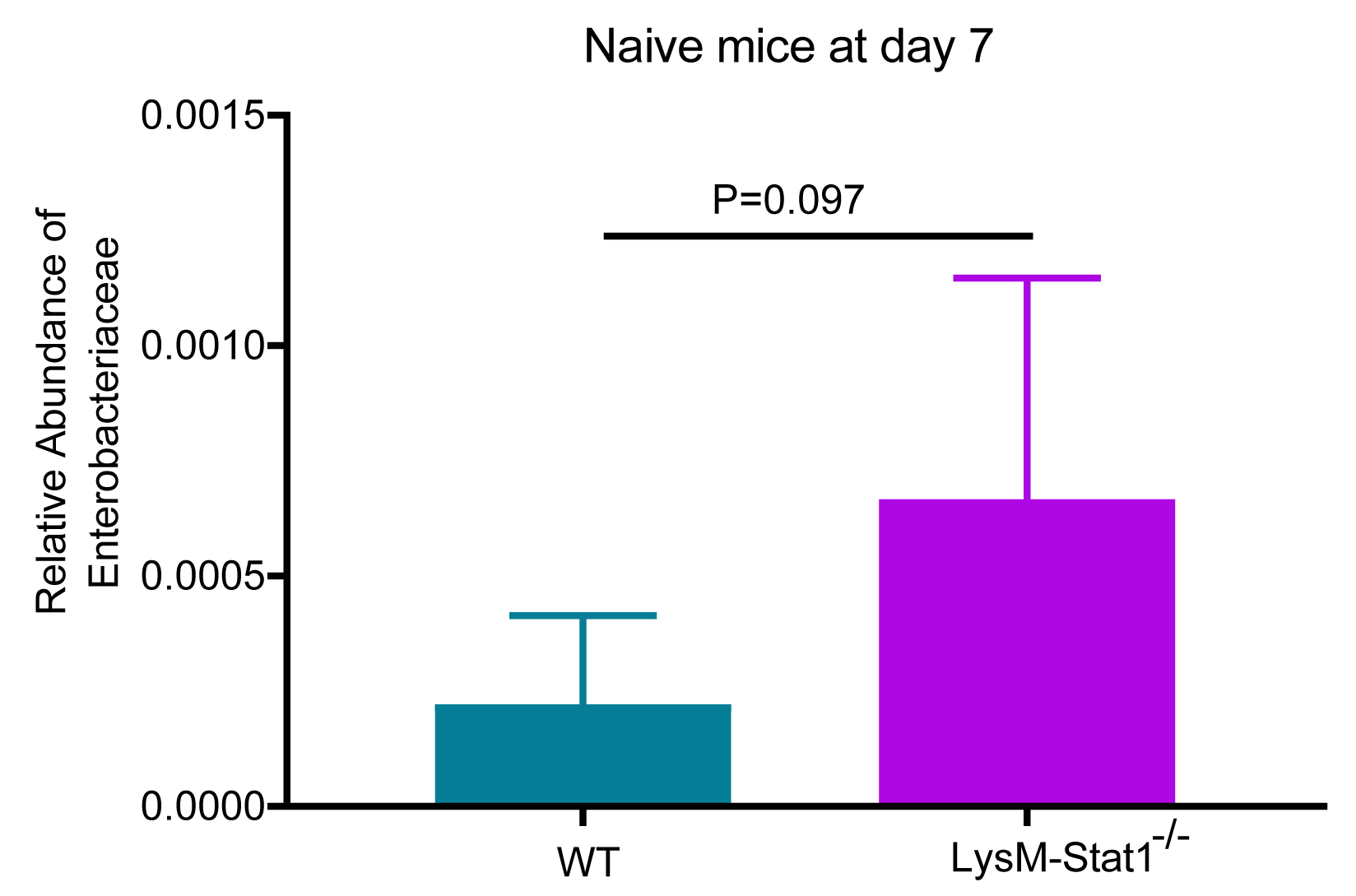

Supplement: FIG S5 [file mBio.00935-19-sf005.tif]
