# Supplementary material for: Infection-Induced Intestinal Dysbiosis Is Mediated by Macrophage Activation and Nitrate Production
Source: mBio. 2019 May 28;10(3):e00935-19. doi: 10.1128/mBio.00935-19 (PMC6538788; doi:10.1128/mBio.00935-19)
Supplement: TABLE S1 [file mBio.00935-19-st001.pdf]

Supplementary File 1. Phyla with differential abundances in naive mice versus *T. gondii* infected mice (day 7) (FDR <0.05).

Phylum	baseMean	log2FoldChange	lfcSE	pvalue	FDR
Verrucomicrobia	2248.253213	-4.512062566	1.323598016	0.000652165	0.003043436
Firmicutes	13675.66782	-1.806365227	0.777674036	0.020191007	0.070668523
Fusobacteria	215.4560698	2.273043736	0.533646939	2.05E-05	0.00014345
Proteobacteria	13886.28784	-5.488306833	0.638562239	8.34E-18	1.17E-16
