# Supplementary material for: Infection-Induced Intestinal Dysbiosis Is Mediated by Macrophage Activation and Nitrate Production
Source: mBio. 2019 May 28;10(3):e00935-19. doi: 10.1128/mBio.00935-19 (PMC6538788; doi:10.1128/mBio.00935-19)
Supplement: TEXT S1 [file mBio.00935-19-s0001.pdf]

Supplementary File 2

Detection of iNOS expression in macrophages of LysM-Stat1$^{-/-}$ mice

Infections with *T. gondii* tachyzoites were performed using the Prugniaud (Pru) strain engineered to express the fluorescent protein tdTomato as previously described (1). These parasites were maintained *in vitro* by serial passage through human foreskin fibroblasts in parasite culture medium (DMEM [Invitrogen, Carlsbad, CA], 20% media M199 [Invitrogen], 10% FBS [Serum Source International, Charlotte, NC], 1% penicillin/streptomycin [Invitrogen], 25 µg/mL gentamicin [Gibco]), and were prepared for infection by serial needle passage and filtered through a 5µm pore filter. Parasites were then rinsed with PBS, and mice (STAT1$^{fl/fl}$ and LysM-Stat1$^{-/-}$) were infected intraperitoneally with 1×10^4 live, invasion competent parasites. The immune response in these mice was examined at 5 days post-infection. Single-cell suspensions were prepared from peritoneal exudate cells (PECs) by a peritoneal wash with 8 ml ice-cold PBS. PECs were then re-suspended in complete RPMI (RPMI with 10% FCS, 1% penicillin-streptomycin, 1 mM sodium pyruvate, 1% nonessential amino acids [Gibco], and 0.1% -mercaptoethanol). Cells for staining (2×10^6) were washed with flow cytometry buffer (PBS, 1% bovine serum albumin [BSA] [Sigma-Aldrich], 2 mM EDTA [Life Technologies]) and blocked in 10 µl Fc block solution (flow cytometry buffer, 1 g/ml anti-CD16/32 2.4G2, 1 g/ml normal rat IgG [Life Technologies]) containing Live/Dead Fixable Aqua dead cell stain (Life Technologies) at 4°C for 10 min. Cells were then stained in 50 µl at 4°C for 15 to 20 min and washed in flow cytometry buffer for acquisition by LSR Fortessa. Antibodies used included anti-CD102-FITC (Biolegend, clone 3C4), anti-CD3-peridinin chlorophyll protein (PerCp)-Cy5.5 (ThermoFisher, clone 145-2C11), anti-CD19-PerCp-Cy5.5 (Biolegend, clone 1D3/CD19), anti-B220-PerCp-Cy5.5 (Biolegend, clone RA3-6B2), anti-NK1.1-PerCp-Cy5.5 (Biolegend, clone PK136), anti-Ly6G-PacBlue (Biolegend, clone 1A8), anti-CD115-Brilliant Violet (BV)-605 (Biolegend, clone AFS98), anti-CD11b-BV650 (Biolegend, clone M1/70), anti-major histocompatibility complex II (MHCII)-BV711 (Biolegend, clone M5/114.15.2), anti-CX3CR1-BV785 (Biolegend, clone SA011F11), anti-NOS2-allophycocyanin (APC) (eBioscience, clone CXNFT), anti-CD11c-APC-R700 (BD Biosciences, clone N418), anti-Ly6C-APC-eFluor780 (ThermoFisher, clone HK1.4), and anti-CD64-phycoerythrin (PE)-Cy7 (Biolegend, clone X54-5/7.1). Results were analyzed using FlowJo 10.1 software.

Bibliography

1. John B, Harris TH, Tait ED, Wilson EH, Gregg B, Ng LG, Mrass P, Roos DS, Dzierszinski F, Weninger W, Hunter CA. 2009. Dynamic Imaging of CD8(+) T cells and dendritic cells during infection with Toxoplasma gondii. PLoS Pathog 5:e1000505.
